# Rethinking Dosing Regimen Selection of Piperaquine for Malaria Chemoprevention: A Simulation Study

**DOI:** 10.1371/journal.pone.0154623

**Published:** 2016-05-16

**Authors:** Nancy C. Sambol, Jordan W. Tappero, Emmanuel Arinaitwe, Sunil Parikh

**Affiliations:** 1 Department of Bioengineering and Therapeutic Sciences, University of California San Francisco, San Francisco, California, United States of America; 2 Centers for Global Health, Centers for Disease Control and Prevention (CDC), Atlanta, Georgia, United States of America; 3 Makerere University School of Medicine, Kampala, Uganda; 4 Yale School of Public Health, New Haven, Connecticut, United States of America; Quensland University of Technology, AUSTRALIA

## Abstract

**Background:**

The combination of short-acting dihydroartemisinin and long-acting piperaquine (DP) is among the first-line therapies for the treatment of uncomplicated *Plasmodium falciparum malaria*. Population pharmacokinetic models of piperaquine (PQ) based on data from acute treatment of young children can be used to predict exposure profiles of piperaquine under different DP chemoprevention regimens. The purpose of our study was to make such predictions in young children.

**Methods:**

Based on a prior population pharmacokinetic model of PQ in young Ugandan children, we simulated capillary plasma concentration-time profiles (including their variability) of candidate chemoprevention regimens for a reference population of 1–2 year olds weighing at least 11 kg. Candidate regimens that were tested included monthly administration of standard therapeutic doses, bimonthly dosing, and weekly dosing (with and without a loading dose).

**Results:**

Once daily doses of 320 mg for three days (960 mg total) at the beginning of each month are predicted to achieve an average steady-state trough capillary piperaquine concentration of 35 ng/mL, with 60% achieving a level of 30 ng/mL or higher. In contrast, weekly dosing of 320 mg (i.e., 33% higher amount per month) is predicted to approximately double the average steady-state trough concentration, increase the percent of children predicted to achieve 30 ng/mL or higher (94%), while at the same time lowering peak concentrations. Exposure at steady-state, reached at approximately 3 months of multiple dosing, is expected to be approximately 2-fold higher than exposure following initial dosing, due to accumulation. A loading dose improves early exposure, thereby reducing the risk of breakthrough infections at the initiation of chemoprevention.

**Conclusions:**

Once weekly chemoprevention of DP predicts favourable exposures with respect to both trough and peak concentrations. These predictions need to be verified, as well as safety evaluated, in field-based clinical studies of young children. Simulations based on prior knowledge provide a systematic information-driven approach to evaluate candidate DP chemopreventive regimens for future trial designs.

## Introduction

Over the past decade, the landscape of malaria therapy has undergone a dramatic shift to the use of artemisinin-based combination therapies (ACTs) as first-line treatment for uncomplicated *Plasmodium falciparum* malaria [1]. These drug regimens have proven to be highly effective, and their use has previously been restricted to the treatment of acute disease. While the short-acting artemisinin “backbone” rapidly reduces parasite burden, a longer-acting partner drug, such as piperaquine (PQ), serves to eliminate residual parasites and reduce the probability that resistance will emerge. Dihydroartemisinin-piperaquine (DHA-PQ; DP), the newest of the World Health Organizations (WHO) first-line recommended regimens for treatment, has been highly efficacious, although recent studies have already demonstrated the emergence of resistance to DHA and PQ in Southeast Asia [1–5].

Concurrent to these developments, DP is under evaluation for chemoprevention, in large part due to the long half-life of PQ (approximately 1 month) [6–8]. Chemopreventive approaches include intermittent preventive treatment (IPT), which uses “therapeutic” doses of antimalarials given at predefined intervals and is directed at the high-risk groups of pregnant women and infants in endemic areas [1]. Seasonal malaria chemoprevention (SMC) uses treatment doses during specific high-risk months [8, 9]. Chemopreventive DP regimens have been highly effective in children from Senegal [10], The Gambia [11], Burkina Faso[8], and Uganda [7, 12, 13]. In a recent study of monthly chemoprevention in Tororo, Uganda, DP was superior to alternative regimens (monthly sulfadoxine-pyrimethamine (SP) or daily trimethoprim-sulfamethoxazole (TS)) [7, 13]. It is likely that higher resistance to the alternative regimens (SP or TS) and the extended half-life of PQ were key determinants of DP’s superior efficacy.

Optimizing the exposure to DP in the preventive setting by improving adherence and fine-tuning the dosing regimen is likewise critical to ensuring its optimal efficacy, limiting its toxicity, and prolonging its useful therapeutic life span [14]. Despite the excellent chemopreventive efficacy of DP, adherence to monthly regimens of three consecutive daily doses of DP is challenging. Evidence from Senegal suggests that only 70% of all children received all 3 doses of IPT each month of the 3-month treatment [10]. A study from Uganda reported that 52% of PQ levels were below the limit of detection at the time malaria was diagnosed and PQ levels did not correlate with time since last reported dose, both of which suggest poor adherence [13]. In the setting of treatment, infants and young children have been shown to have lower DP exposure which correlates with higher rates of recurrent infection than in older children and adults [6, 15–17]. As a result of these and other pharmacokinetic (PK) and pharmacodynamic (PD) studies of DP, recent 2015 WHO guidelines have recommended higher treatment doses of DP, particularly in young children (Table 1) [1].

**Table 1:** DP weight-based dosing guidelines.

Body Weight (kg)	Recommended Dose (mg)	
	Former WHODP dosing regimen[22]	Revised WHO2015 DP dosing regimen[1]
**5 to 7**		160
**8 to 10**	160	240
**11 to 14**	240	320
**15 to 16**	320	320
**17 to 19**	320	480
**20 to 23**	400	480
**24**	480	480

Significantly less attention has been focused on characterizing the exposure of DP with chemoprevention. A study in Thai adults found that monthly DP was superior to bimonthly DP for preventing uncomplicated malaria, likely due to a decline in PQ concentrations below a protective threshold in the second month after dosing with the latter [18]. In this study, all subjects with recurrent infection had trough venous plasma concentrations below 32 ng/mL. Similarly, in a Ugandan monthly DP chemoprevention study in children, PQ exposure was strongly predictive of protective efficacy [19]. On the other hand, a recent adult DP chemoprevention study was halted due to concerns of QT prolongation associated with high peak PQ levels [20].

Assuming that a threshold concentration for effective preventive treatment exists, and that PK determines the profile of systemic exposure, we hypothesized that known (prior) PK can inform dosing of PQ-containing preventive regimens to optimize efficacy and limit toxicity. In the study reported herein, we use a population PK model from a study of DP treatment in children ages 6 months to 2 years in the high transmission area of Tororo, Uganda [6, 15] to simulate PQ concentration-time profiles with various DP preventive regimens.

## Materials and Methods

### Pharmacokinetic Model

Data were simulated based on a previously published model for the same population [6]. Briefly, the model was derived from a population PK study of 107 children 6 months to 2 years of age from Tororo, Uganda, that was part of a larger clinical trial comparing the efficacy of artemether-lumefantrine to DP for treatment of uncomplicated malaria [15, 21]. DP was administered to children with food as a single treatment of 3 daily doses (20/160 mg DHA/PQ per dose for patients weighing 5.1–10.4 kg, and 30/240 mg DHA/PQ per dose for those weighing 10.5–14.5 kg), according to 2010 World Health Organization Malaria Treatment Guidelines (Table 1) [22]. Capillary plasma samples for PK analysis were obtained during each episode (with an average 2 episodes per child). Population PK models were constructed using mixed effects modeling with the program NONMEM^®^ [23]. The final model was a three-compartment open model with first-order absorption, unconditional allometric scaling of each of the three clearance and three volume of distribution parameters and a statistically significant age effect on *CL*/*F* [6]. The final model for *CL*/*F* was as follows:

$$\tilde{CL/F}\text{ [L/h]}=\;6.39\;\cdot \;{\left(\frac{\text{W}{\text{T}}_{i}}{8.36}\right)}^{0.75}\cdot \;{\left(\frac{\text{AG}{\text{E}}_{i}}{12}\right)}^{0.35},$$ (1)

in which WT*i* is the body weight (kg) and AGE*i* the age (month) of the *i*th child. Weight was centered at 8.36 kg (the approximately median value of a 1 year-old in this population), and age at 12 months for ease of interpretation.

### Population PK Simulations

Simulations of data sets with 2000 hypothetical 1–2 year olds weighing ≥ 11 kg were generated (referred to as the reference population). This population was selected, rather than simulate all ages/weight groups, for purposes of efficiency. Assuming that the ratio of doses in the preventive setting is similar to that in the acute setting, one can infer the required dose in non-simulated groups from that of the simulated reference group. To mimic the original data, a linear regression model was used to simulate weight [6]. This model included variability and a correlation (*r*^2^) between age and weight of 0.21. An alternate method (not available at the time the simulation study began), would be to use published regional weight-for-age charts [24]. The first 2000 individuals with the combined age and weight that met the prescribed criterion were included in the capillary plasma concentration simulations. The program NONMEM^®^, Version 7.3 (ICON, Dublin, Ireland) [23] generated the predictions and the program R (version 2.15.0) was used to create the plots. Administered doses and PK were assumed to remain constant within an individual and we assumed the absence of any drug interaction as well as the absence of a disease (active malaria infection) effect. Considering that the drug may be used continuously in some settings, PK descriptors of particular interest included PQ capillary plasma trough concentration during the first 5 months (the time during which concentrations are increasing) and at steady-state (after 12 months of dosing). In addition, we summarized peak concentrations at steady-state and after a loading dose, overall concentration-time profile at steady-state, and overall concentration-time profile during the first month with and without a loading dose. For purposes of this study, a loading dose was defined as the standard three consecutive daily dose regimen.

The regimen that served as a point of reference was 240 mg (standard dose for acute treatment in those weighing 10.5 to 14.5 kg per WHO 2010 guidelines) given daily for 3 days at the beginning of each month, as this schedule has been most studied in treatment and prevention trials [22]. An increased dosing regimen of 320 mg (new recommended dose for acute treatment in those weighing 11–16 kg per 2015 WHO guidelines) [1] was also simulated, as well as increasing the total dose *level* further by 25%, varying the dosing *frequency* [monthly, bimonthly (twice monthly), and weekly] and administering a *loading dose*.

Given that precise target PQ concentrations in different endemic and demographic settings are still being evaluated in the setting of treatment, and with minimal data available in chemoprevention settings, we chose to focus on putative target capillary plasma trough (prior to each dose) steady-state concentrations of 10, 20 and 30 ng/mL, allowing one to compare exposure levels between different approaches. A trough PQ venous plasma level of 32 ng/mL was found to be associated with the risk of breakthrough malaria in Thai adults given DP chemoprevention [18]. Additionally, a day 7 capillary plasma level of 57 ng/mL (equating to a venous plasma level of 30 ng/mL) was selected as a target-of-interest, based on data from treatment studies showing that this threshold is predictive of the risk of recurrent malaria [17, 25, 26].

## Results

### Effects of changing DP dose level on piperaquine exposure

**Fig 1:**
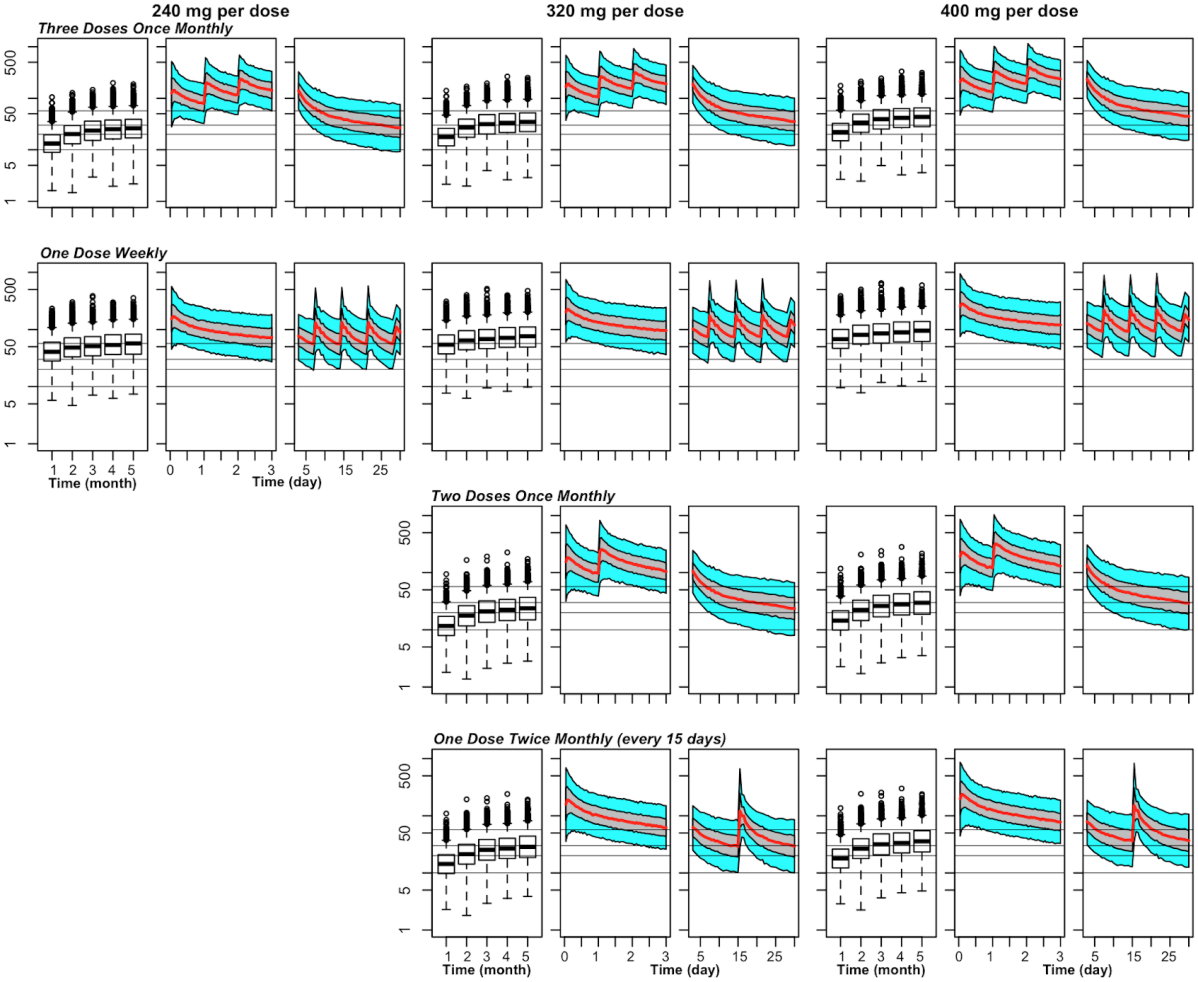
Predicted capillary plasma PQ concentration versus time for various chemoprevention regimens of DP in 1–2 year olds weighing ≥ 11 kg. For each combination of dose (240 mg, 320 mg, or 400 mg) and schedule (3 times once monthly, once weekly, 2 times once monthly or once every 15 days), there is a set of 3 panels, with the left panel (box plots) showing trough concentrations at the end of the first 5 months, the middle panel the steady-state PK profile during the first 3 days after the first dose of the month, and the right panel the steady-state PK profile during days 3–30 (or days 3–28 for weekly dosing) relative to a first dose of the month. For the PK profiles, the middle line represents the population predictions, the grey shaded area the 50% prediction interval and the outer bounds the 90% prediction interval. The grey horizontal lines indicate capillary plasma concentrations of 10, 20, and 30 ng/mL (for putative trough targets), and a capillary plasma level of 57 ng/mL (for a putative day 7 target).

Fig 1 and Table 2 show the effects on PQ exposure seen with various changes in the DP regimen, including an increase in dose, while maintaining a consistent frequency. An increase in monthly PQ dose from 240 mg to 320 mg (given once daily x 3) was associated with a predicted PQ trough steady-state capillary plasma concentration that increased from 27 to 35 ng/mL. With an increase to 400 mg per dose, predicted PQ trough steady-state capillary plasma concentrations increased to 44 ng/mL. While all doses tested are predicted to achieve trough capillary plasma concentrations of 10 ng/mL in 93% or more individuals, differentiation among doses widens with target trough levels of 20 ng/mL and 30 ng/mL. Additionally, as compared with the 240 mg dose, a 50% increase in the number of individuals predicted to achieve day 7 levels of above 57 ng/mL is expected with a 400 mg dose.

**Table 2:** Predictions related to trough and peak capillary plasma concentration of PQ at steady-state with various chemoprevention regimens of DP in 1–2 year olds weighing ≥ 11 kg.

Dose	Frequency	Predicted Trough (ng/mL)		Percent of Patient-Treatments with Predicted Capillary Concentration above Given Level				Predicted Maximum Concentration (ng/mL)	
				Day 30 (Trough)*			Day 7		
		Geometric Mean	90% prediction interval	10 ng/mL	20 ng/mL	30 ng/mL	57 ng/mL	Geometric Mean	90% prediction interval
240 mg daily x 3	monthly	26.5	9.2, 75.7	93.3	66.8	42.8	58.3	408.2	185.9, 897.2
240 mg x 1	weekly	55.2	20.6, 147.2	99.7	95.3	83.7	NA	303.7	120.0, 722.3
320 mg daily x 3	monthly	35.3	12.2, 108.9	97.1	80.7	60.2	76.3	544.3	247.9, 1196.4
320 mg daily x 2	monthly	23.3	8.0, 66.6	89.5	59.2	36.4	45.3	441.5	173.7, 1007.9
320 mg x 1	twice monthly	29.1	10.1, 85.4	95.1	72.7	48.2	NA	334.7	124.6, 860.8
320 mg x 1	weekly	73.6	27.5, 196.2	100	98.1	93.5	NA	404.9	172.1, 963.1
400 mg daily x 3	monthly	44.2	15.3, 126.1	98.8	87.8	72.1	86.8	680.1	309.8, 1495.5
400 mg daily x 2	monthly	29.1	10.0, 83.2	95.1	71.2	48.9	60.6	551.9	217.1, 1259.8
400 mg x 1	twice monthly	36.3	12.6,106.7	97.8	82.9	62.4	41.6	418.4	155.8, 1076
400 mg x 1	weekly	92.0	34.3, 245.2	100	99.3	96.4	NA	506.1	215.0, 1203.9

*Day 30 except for weekly administration, in which case it is day 28

NA = not applicable

Current chemoprevention trials have studied regimens of three consecutive doses given daily each month, though adherence to this regimen is suboptimal [10, 13]. An alternative approach that may improve adherence is to either reduce the number of consecutive doses (two doses daily each month), or increase the frequency of dosing to either twice monthly/every two weeks or weekly (below). Reducing the number of consecutive doses to 2 doses while increasing the amount in each dose (to 320 mg or 400 mg) leads to predicted exposure that is comparable to the reference regimen (Fig 1 and Table 2).

### Effects of changing the dosing frequency on piperaquine exposure

Increasing the dosing frequency to twice monthly also leads to steady-state trough concentrations comparable to those of the reference. Compared to the reference regimen, 320 mg twice monthly achieves similar steady-state trough concentrations, while 400 mg twice monthly leads to a 36% increase in steady-state trough concentrations and a 46% increase in predicted percent of individuals with troughs above 30 ng/mL. A further increase in the dosing frequency to single weekly doses results in additional improvement in PQ trough exposure. Compared to the reference regimen, weekly 240 mg, 320 mg, and 400 mg doses increase the steady-state trough concentration by 2-fold, 2.8-fold, and 3.5-fold, respectively. In addition, even with a 240 mg dose, >99% and >84% of individuals are predicted to have trough concentrations above 10 ng/mL and 30 ng/mL, respectively.

### Effects of dosing changes on peak plasma piperaquine concentration

While no clear threshold peak concentration has been linked with toxicity, a recent trial reported that PQ exhibited a concentration-dependent effect on QT prolongation. Adults whose PQ venous plasma peaks were in the range of approximately 500 to 1000 ng/mL had a higher risk of cardiotoxicity [27]. Given this potential relationship, capillary plasma peaks were evaluated in our simulation study (Fig 1 and Table 2). While more frequent dosing results in higher troughs, mean predicted peak PQ capillary plasma concentration is substantially lower, going from 544 ng/mL to 340 ng/mL with monthly (x 3) versus once weekly dosing of 320 mg, respectively, despite higher total monthly doses with the later. Similar reductions in predicted peak concentrations are noted with the weekly 240 mg and 400 mg regimens. Peaks with the bimonthly regimens are likewise lower, in part due to the lower total monthly doses.

### Effects of loading dose on piperquine exposure

**Fig 2:**
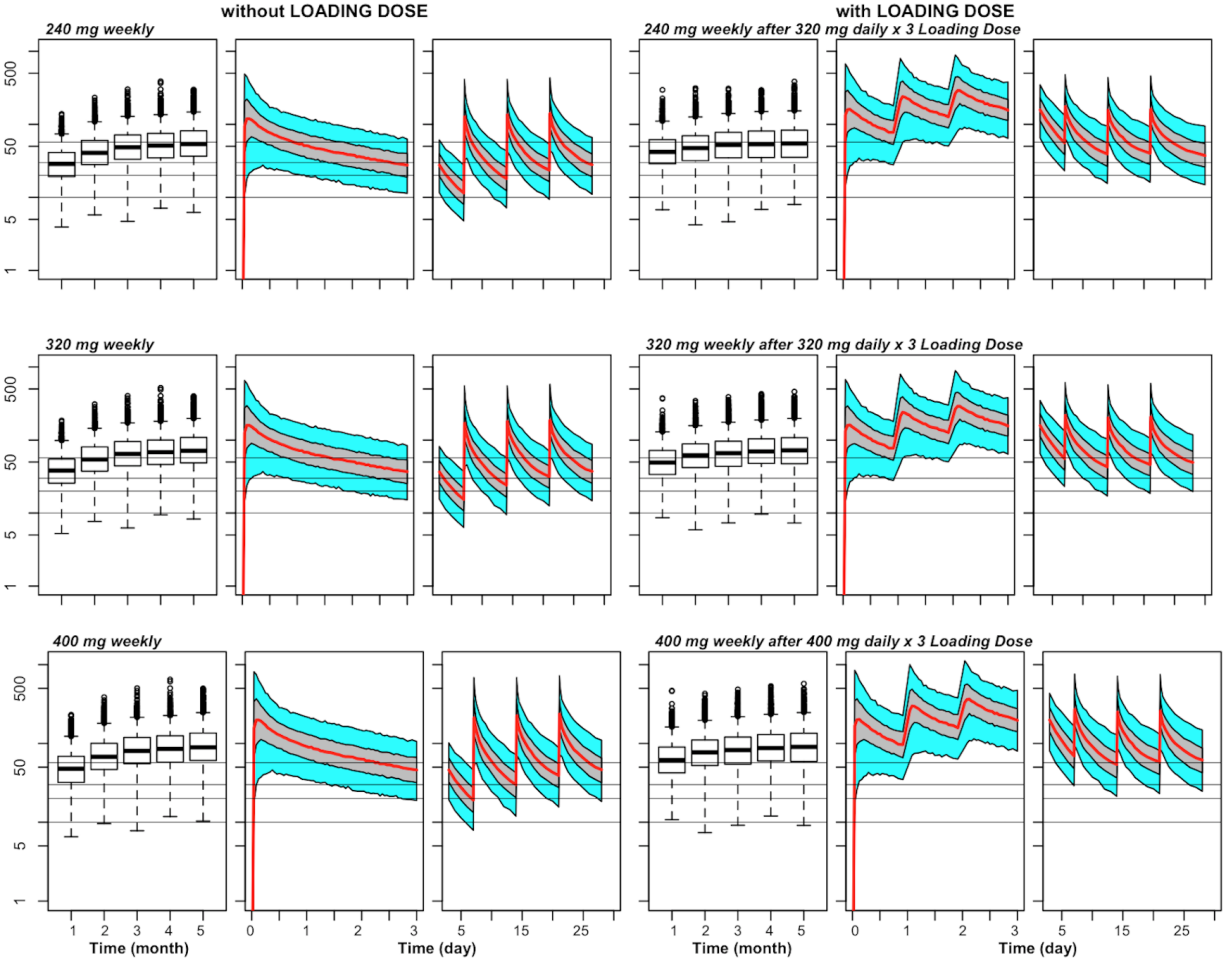
Predicted capillary plasma PQ concentration versus time for different doses of weekly chemoprevention regimens of DP without and with a loading dose in 1–2 year olds weighing ≥ 11 kg. For each combination of dose (240 mg, 320 mg, or 400 mg given weekly) and presence or absence of a loading dose, there is a set of 3 panels, with the left panel (box plots) showing trough concentrations of the first 5 months, the middle panel the PK profile during the first three days after the first dose, and the right panel the PK profile during days 3–28 relative to the first dose. For the PK profiles, the middle line represents the population predictions, the grey shaded area the 50% prediction interval and the outer bounds the 90% prediction interval. The grey horizontal lines indicate capillary plasma concentrations of 10, 20, and 30 ng/mL (for putative trough targets), and a capillary plasma level of 57 ng/mL (for a putative day 7 target).

Simulations revealed that PQ capillary plasma concentrations with repeated dosing are expected to reach steady-state in approximately three months (Figs 1 and 2). The PQ steady-state concentrations after multiple treatments relative to with a single treatment are approximately doubled due to accumulation. For example, after 3 consecutive daily doses of 240 mg, the average (geometric mean) predicted PQ trough capillary plasma concentration at the end of the first month is 13.2 ng/mL (95% prediction interval 5.1, 34.1 ng/mL), compared to steady-state trough levels of 26.5 ng/mL (95% prediction interval 9.2, 75.7 ng/mL) if this dose is given monthly. To address the reduced exposure in early months, we simulated the impact of a loading dose (three consecutive daily doses at the beginning of the first dosing month), followed by weekly dosing (Fig 2 and Table 3). The use of a 320 mg daily x 3 loading dose, followed by 240 mg weekly dosing, is predicted to increase trough levels to 55.2 ng/mL at the end of the first month (approximately the same value as the trough at steady-state), as compared to 41.2 ng/mL without a loading dose. Similarly, the use of this loading dose followed by a 320 mg weekly dose led to an 32% increase in the first-month trough level as compared to using the 320 mg weekly dose without a loading dose.

**Table 3:** Comparative predicted capillary plasma PQ concentrations with and without a loading dose of DP and predicted maximum concentration with a loading dose of DP in 1–2 year olds weighing ≥ 11 kg.

Maintenance Dose	Loading Dose	Predicted Ratio of Day 7 Concentration after First Dose to Day 7 Concentration at Steady-State		Predicted Ratio of Day 28 Trough to Steady-State Trough		Predicted Maximum Concentration on Day 3	
		Without Loading Dose	With Loading Dose	Without Loading Dose	With Loading Dose	Geometric Mean	90% prediction interval
240 mg weeklyweekly	320 mg daily x 3	0.75 (0.20, 2.85)	1.01 (0.27, 3.80)	0.50 (0.13, 1.86)	0.75 (0.19, 2.78)	494.3	200.6, 1116.3
320 mg weeklyweekl	320 mg daily x 3	0.21 (0.06, 0.79)	0.75 (0.20, 2.85)	0.50 (0.13, 1.86)	0.66 (0.17, 2.49)	494.3	200.6, 1116.3
400 mg weekly	320 mg daily x 3	0.21 (0.06, 0.79)	0.75 (0.20, 2.85)	0.50 (0.13, 1.87)	0.66 (0.17, 2.49)	617.9	250.8, 1395.5

## Discussion

A growing body of evidence supports the efficacy of monthly chemoprevention with DP in various epidemiologic settings [10–13, 18, 28]. While such an approach has been quite effective in tightly controlled research studies, challenges with adherence to monthly regimens, the emergence of resistance to DP in Southeast Asia, and concerns over the risk of cardiotoxicity with repeated monthly treatment doses [20] warrant characterization of the profile of DP with alternative chemoprevention regimens. The objective of this PK simulation study was to apply population PK model-derived information to predict exposure with alternative DP chemoprevention dosing regimens in young children. As precise PQ target levels for DP chemoprevention remain unclear, we simulated steady-state troughs (end of the month), day 7 concentrations (which are associated with the risk of recurrent infection after treatment), and peak concentrations (which may be predictive of toxicity) under differing dosing scenarios. Our results suggest that weekly single dose DP will lead to higher steady-state trough PQ concentrations, as well as the proportion of individuals reaching specified trough levels, while at the same time lowering the peak concentrations. Furthermore, the use of a loading dose is predicted to attain concentrations in the first several months following initiation of chemoprevention that are more in line with those seen at steady-state.

Amongst our initial simulations was a comparison of previous mg/kg DP dosing regimens to the newly revised increased 2015 WHO regimen, as all published chemoprevention studies have been performed under earlier mg/kg dosing regimens (Table 1) [1, 22]. Relative to the former 240 mg dose (daily x 3 given monthly), the revised 320 mg per dose regimen in young children is predicted to provide a 38% increase in steady-state troughs with 60% of children maintaining capillary troughs above 30 ng/mL, as compared to 43% under prior dosing weight bands. These results are consistent with a recent simulation study utilizing the Thai adult chemoprevention data which found that the newly revised WHO dosing regimen provides a significant improvement in efficacy in all weight groups, but particularly in those weighing 8 to 12 kg [18, 29].

While it is anticipated that current increased DP mg/kg dosing guidelines will improve PK exposure in both treatment and chemopreventiion settings, adherence to the regimen for the latter, with three consecutive daily doses each month, is likely to be challenging [13, 30]. A recent study in a high transmission region of Uganda found that while DP was the most efficacious of chemopreventive regimens studied, it was still only 58% effective with 52% of children having undetectable PQ at the time of diagnosis, suggesting a lack of adherence [13]. It is possible that the provision of single doses at a regular interval (particularly weekly) may improve adherence over a regimen that is administered for three consecutive days of each month, though this remains to be evaluated in field settings. Our simulated regimens thus included single dose twice monthly and weekly administration which may provide simplified dosing at regular intervals. Most promising was the provision of weekly chemoprevention, with an associated 2- to 3.5-fold increase in steady-state trough concentrations compared to monthly dosing. Notably, mean steady-state trough concentrations with all weekly doses are predicted to be higher than the protective venous trough level of 20 ng/mL reported recently (which is predicted to correspond to a capillary level of approximately 50 ng/mL) [17, 29]. Splitting a total dose into two or more fractional doses (and giving more frequently) has the additional benefit that if a dose is missed, it does not have as much impact on the average systemic exposure [31]. If the same (or even greater) percentage of pills are missed overall relative to monthly dosing, dividing a similar total monthly dose into multiple doses throughout the month is still likely to result in higher PQ trough concentrations. In a trial comparing once daily to twice daily antiretroviral therapy, the probability of sustained virologic response was higher (89% versus 76%) with the latter, despite *less* adherence (80% versus 91%) because trough levels of the antiretroviral lopinovir were higher [32].

Another potential weakness of current chemopreventive approaches is the time that is required to reach steady-state. With the provision of three consecutive daily doses, steady-state is projected to be achieved after a few months of dosing, placing individuals at a higher risk of breakthrough infections in the first few months after initiation. In the recent Thai adult study, 4 of 5 new infections occurred during the first 2 months of the trial before steady-state was achieved [18]. Additionally, 32 of the 40 infections during the every-2-month DP arm occurred in the second month after a dose, also supporting the notion that target concentrations need to be maintained for the duration of the dosing interval for optimal efficacy. To address this concern, we simulated the use of a loading dose (the standard three consecutive daily dose regimen), followed by a repeated weekly single 320 mg dose regimen (Table 3, Fig 2). The use of a loading dose is predicted to increase PQ exposure closer to steady-state in the first few months, as compared to weekly dosing without the loading dose. Levels of PQ on day 7 of the 1^st^ month are expected to be 75% of steady-state with the use of a loading dose as compared to 21% of steady-state without a loading dose. Improved exposure in the first two months of chemoprevention is particularly critical in high transmission settings and in the setting of SMC, where DP is only administered for a few months of the year.

An obvious concern with certain alternative regimens is that of potentially greater toxicity, the most serious of which is corrected QT (QTc) interval prolongation [33]. Most notably, a recent trial was halted due to concerns of QT prolongation when a compressed monthly 2-day treatment was attempted in healthy adults, and that toxicity was felt to relate to PQ peak concentrations [20]. Importantly, our simulations suggest that weekly dosing *decreases* predicted peak levels, despite higher steady-state trough concentrations (Tables 2 and 3). In other words, our simulations suggest that *both* improved efficacy and lower risks of toxicity may be achieved with this approach.

An additional concern that one must consider with repeated dosing in chemoprevention is the potential for the emergence and selection of resistance [34]. Resistance to both artemisinins and PQ has been described in Asia, and the potential emergence or spread of resistance in Africa is of great concern [3–5, 35–37]. While we did not model PK-resistance relationships in this study, regimens that provide higher steady-state troughs are those with shorter dosing intervals, resulting in concentrations that are more likely to be above a “protective” threshold where the development or selection for resistance may occur. Further, the use of twice monthly or weekly doses may be associated with “insufficient” exposure to PQ after the first dose(s), as compared with levels after a standard “treatment” dose, thereby placing one at risk for selection or emergence of resistant PQ parasites. The use of a “loading dose” at the start of chemoprevention improves the likelihood of parasite eradication in asymptomatically-infected individuals, thereby reducing this potential risk.

An additional challenge in our PK simulation study was to define a target “protective” PQ concentration, as a clear target has not been described for chemoprevention. While data are emerging [18, 29], multiple factors such as transmission intensity, presence of artemisinin or partner drug resistance, immunity of the population, age (adults vs children), and pregnancy status are likely to impact protective thresholds. In the absence of such PK/PD data, we chose to evaluate several PQ targets at troughs and at day 7 to allow for comparatives PK exposure profiles among dosing approaches. In the future, a fully systematic approach would be ideal, combining known PK models with mechanistic disease and drug-disease (i.e., PD) models to predict response under appropriate conditions and assumptions. Such mechanistic models for malaria and its treatment are being developed [29, 38–44].

A limitation of the current study is our focus on the long-acting partner drug PQ, without simulating exposure to the short-acting artemisinin, DHA. Most notably, the PD impact of altered DHA exposure under modified dosing regimens (bimonthly, weekly) is an important consideration. Currently, no PK data for artemisinins is available from chemoprevention studies, though a recent study has modeled DHA exposure and parasite killing rates under differing DP treatment regimens [45]. In addition, our study uses PK information from a diseased population to make predictions in a non-diseased population. While alterations in PK in the presence of malaria have been identified for some antimalarials, namely lumefantrine, [46] artusenate, [47–49] and halofantrine, [48], such data for PQ are limited, though some alteration in PK almost certainly is to be expected. Of note, a significant effect of baseline parasite density on drug clearance or relative bioavailability was not detected in the PQ data from which our model was derived [6].

In summary, we believe that this or similar simulation approaches can be very beneficial in choosing dosing strategies for chemoprevention trials of DP and other antimalarial regimens. Our simulations predict that, in young children receiving a weekly single dose of DP, excellent exposure can be maintained throughout the dosing interval while lowering peak PQ concentrations, compared to current monthly “treatment” doses. Weekly dosing is also expected to maintain higher troughs when adherence is imperfect. The use of a loading dose may further enhance the potential for success through the “complete” eradication of parasites and reduction in breakthrough infections during the first month. It is critical that the impact of the predicted exposure (average, trough and peak) be considered in the context of patient- and regional-specific requirements for efficacy, toxicity, ease of administration, adherence, and risk of resistance.
